# PSA kinetics before 40 years of age

**DOI:** 10.1590/S1677-5538.IBJU.2017.0710

**Published:** 2018

**Authors:** Cristiano Linck Pazeto, Thiago Fernandes Negris Lima, José Carlos Truzzi, Nairo Sumita, José de Sá, Fernando R. Oliveira, Sidney Glina

**Affiliations:** 1 Departamento de Urologia, Faculdade de Medicina do ABC, Santo André, SP, Brasil; 2 Fleury Medicina e Saúde São Paulo, SP, Brasil; 3 Departamento de Epidemiologia, Universidade de São Paulo, São Paulo, SP, Brasil

**Keywords:** Prostate-Specific Antigen, Kinetics, Prostatic Neoplasms

## Abstract

**Purpose:**

The baseline PSA has been proposed as a possible marker for prostate cancer. The PSA determination before 40 years seems interesting because it not suffers yet the drawbacks related to more advanced ages. Considering the scarcity of data on this topic, an analysis of PSA kinetics in this period seems interesting.

**Materials and Methods:**

A retrospective assay in a database of a private diagnostic center was performed from 2003 to 2016. All subjects with a PSA before 40 years were included.

**Results:**

92995 patients performed PSA between the ages of 21 – 39. The mean value ranged from 0.66 ng / mL (at age 22) to 0.76 ng / mL (at age 39) and the overall mean was 0.73 ng / mL. As for outliers, 3783 individuals presented a baseline PSA > 1.6 ng / mL (p95). A linear regression model showed that each year there is a PSA increase of 0.0055 ng / mL (β = 0.0055; r^2^ = 0.0020; p < 0.001). A plateau in PSA between 23 and 32 years was found and there were only minimal variations among the ages regardless of the evaluated percentile.

**Conclusion:**

It was demonstrated that PSA kinetics before 40 years is a very slow and progressive phenomenon regardless of the assessed percentile. Considering our results, it could be suggested that any PSA performed in this period could represent the baseline value without significant distortions.

## INTRODUCTION

Baseline prostate-specific antigen (PSA) has been proposed as a possible marker to detect those who would be at increased risk for developing prostate cancer. The concept of baseline PSA began when Gann et al. (1995) showed the role of this test as a predictor of prostate cancer in men with PSA > 1.0 ng / mL at a median age of 62.9 (1). Similarly, in a subgroup of The European Randomized study of Screening for Prostate Cancer (ERSPC), subjects with baseline PSA > 1.0 ng / mL and > 2.0 ng / mL had an increased hazard ratio for prostate cancer specific mortality (4.0 - fold and 7.6 - fold respectively) compared with those who had < 1.0 ng / mL levels (2). Regarding PSA in young adulthood, a study enrolling 325 men have demonstrated that the fourth quartile of baseline PSA (0.56 ng / mL) was associated with an increased odds of prostate cancer before age 65 (3). Additionally, Lilja et al. reported the largest association between baseline PSA and subsequent prostate cancer in those with 40 years (4). Despite this, data on this topic is scarce which justifies more studies. Thus, we aim to determine the PSA kinetics before 40 years.

## MATERIAL AND METHODS

A retrospective assay in Fleury^®^ institute database was performed to determine how many subjects had measured the PSA between ages 21 and 39. The Fleury^®^ institute is a private diagnostic center represented by a conglomerate of 33 laboratory units in Sao Paulo, Brazil. We highlight however that this is not a specific center for cancer diagnosis and the tests are usually performed by request of private clinics and companies. Clinical data were not available.

Considering the same PSA ultra - sensitive kit, all samples from 2003 to 2016 were included. In cases of repeated dosages for the same subject, only the first PSA was included in the study. The PSA values > 4.0 ng / mL were analyzed separately for the major association with prostate pathologies such as prostatitis and prostate cancer. Measures of central tendency, variability (mean and standard deviation), median, confidence interval, values range and percentiles were used for quantitative variables. The distribution of data was verified with Shapiro - Wilk test. For PSA age - specific levels comparisons, the Kruskal - Wallis with a Dunn's post - test was applied. The Spearman's test was used for correlation analysis of “PSA” and “Age”. Finally, a linear regression analysis considering dependent variable “PSA” and independent variable “Age” was included. Due to the non - normality of the “PSA” variable, the logarithm transformation method was performed.

After this first analysis, we divided the individuals in two groups: “Group 1”, with men who performed ≥ 2 dosages and “Group 2” with those who performed dosage only once. Again, only the first PSA was considered and an analysis of mean, median and percentiles was performed for each age in both groups. Additionally, the Chi - Square test was applied to compare the number of men with PSA > 1.0 ng / mL in both groups. The statistical analysis was performed using STATA version 12.0 with a level of significance of 5%.

## RESULTS

During the period analyzed (2003 – 2016), 92.995 men performed a PSA between 21 – 39 years (Baseline Group). A total of 128.948 dosages were accounted. There were 32.721 men who repeated the PSA (Group 1) and 60.274 who did not (Group 2).

**Figure 1:**
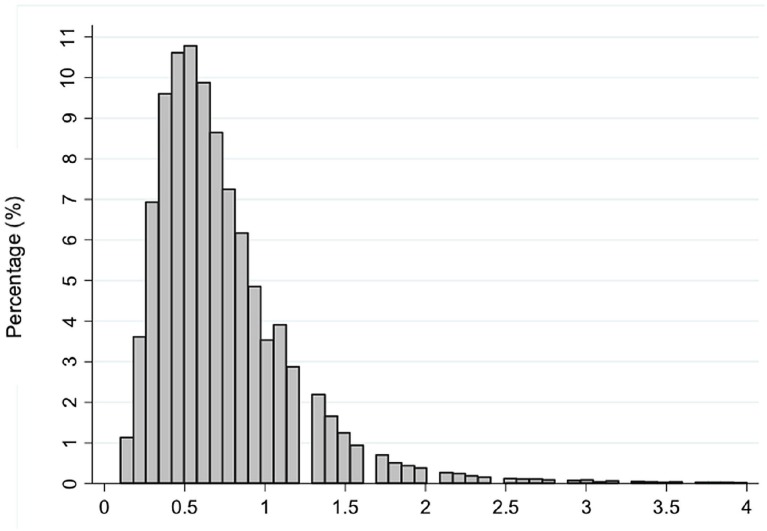
Distribution of PSA values.

**Figure 2:**
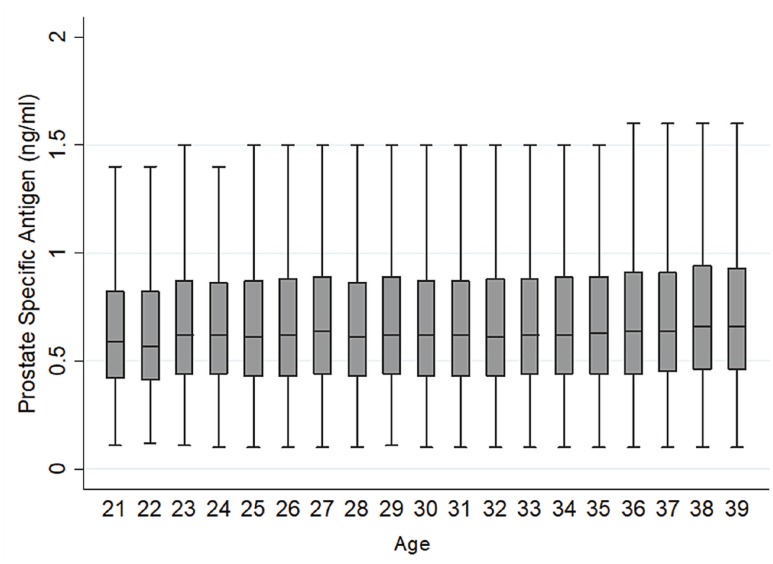
Box plot of PSA according to age.

Considering the “Baseline Group” (Table-1), the most common age was 39 years with 14.525 men (15.62%). The mean PSA value ranged from 0.66 (at age 22) to 0.76 ng / mL (at age 39) and the overall mean was 0.73 ng / mL ± 0.45. Most values remained between 0.3 – 1.0 ng / mL (Figure-1). As for outliers, we found 3783 individuals with a baseline PSA > 1.6 ng / mL (p95) distributed among all ages (Figure-2).

**Table 1:** Descriptive analysis of total PSA according to age.

Age	PSA						
	n (%)	Mean (SD)	Median	Min	Max	Percentiles 25;75	Percentiles 90;95
21	800 (0.86)	0. 683 (0.42)	0.60	0.1	3.8	0.42; 0.82	1.2; 1.4
22	956 (1.03)	0.664 (0.37)	0.57	0.1	3.6	0.41; 0.82	1.1; 1.3
23	1112 (1.21)	0.718 (0.43)	0.62	0.1	4.0	0.44; 0.87	1.2; 1.5
24	1265 (1.36)	0.711 (0.43)	0.62	0.1	4.0	0.44; 0.86	1.2; 1.5
25	1531 (1.65)	0.720 (0.46)	0.61	0.1	3.9	0.43; 0.87	1.2; 1.5
26	1829(1.97)	0.721 (0.45)	0.62	0.1	3.8	0.43; 0.88	1.2; 1.5
27	2099(2.26)	0.717 (0.41)	0.64	0.1	3.6	0.44; 0.89	1.2; 1.5
28	2593 (2.79)	0.713 (0.44)	0.61	0.1	4.0	0.43; 0.86	1.2; 1.5
29	3106 (3.34)	0. 720 (0.44)	0.62	0.1	3.9	0.44; 0.89	1.2; 1.5
30	3106 (4.34)	0. 720 (0.44)	0.62	0.1	4.0	0.43; 0.87	1.2; 1.5
31	4459 (4.79)	0. 712 (0.44)	0.62	0.1	3.9	0.43; 0.87	1.2; 1.5
32	5293 (5.69)	0.711 (0.42)	0.61	0.1	3.9	0.43; 0.88	1.2; 1.5
33	5942 (6.39)	0. 722 (0.43)	0.62	0.1	4.0	0.44; 0.88	1.2; 1.5
34	6796 (7.31)	0. 727 (0.45)	0.62	0.1	4.0	0.44; 0.89	1.2; 1.5
35	7682 (8.26)	0. 734 (0. 44)	0.63	0.1	4.0	0.44; 0.89	1.2; 1.6
36	8579 (9.23)	0. 741 (0. 45)	0.64	0.1	4.0	0.44; 0.91	1.2; 1.5
37	9457 (10.17)	0. 748 (0. 46)	0.64	0.1	4.0	0.45; 0.91	1.3; 1.6
38	10925 (11.75)	0. 768 (0.47)	0.66	0.1	4.0	0.46; 0.94	1.3; 1.6
39	14525 (15.62)	0. 764 (0.46)	0.66	0.1	4.0	0.46; 0.93	1.3; 1.6
Total	92995 (100)	0. 737 (0.45)	0.63	0.1	4.0	0.44; 0.90	1.3; 1.6

**Figure 3:**
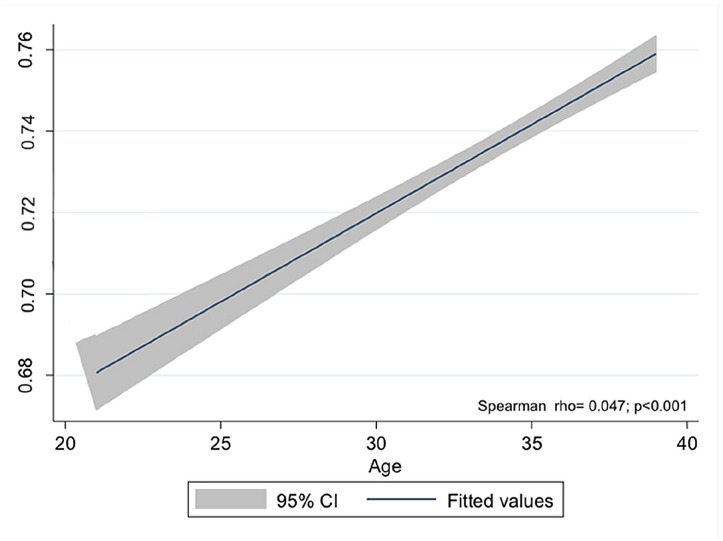
Correlation between PSA values and ages.

**Figure 4:**
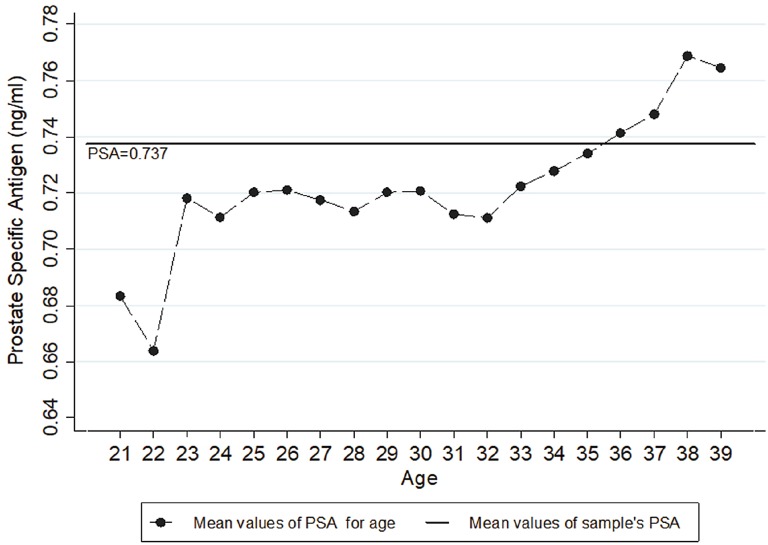
Graphic model of the mean values of PSA according to age.

**Figure 5:**
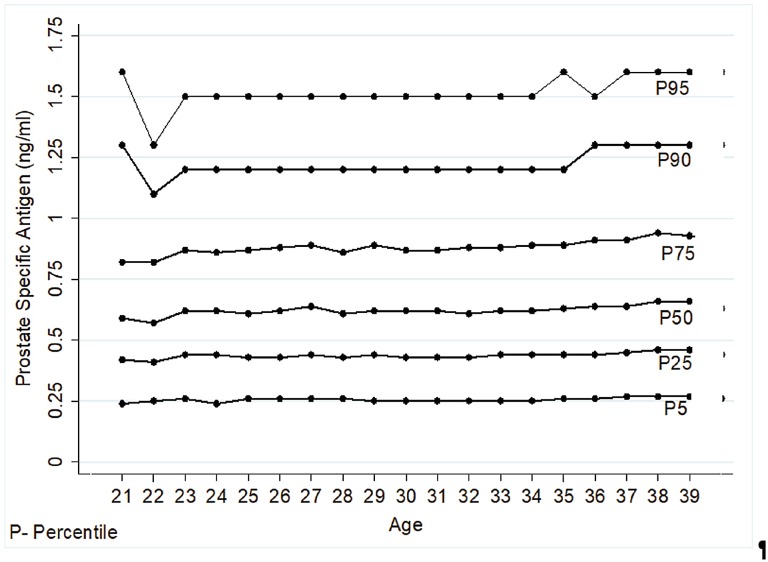
PSA percentiles values according to age.

The annual PSA variation was not statistically significant, except for the ages 22 – 23, 34 – 35 and 37 – 38 (Table-2). However, there was a positive correlation between “PSA” and “Age” in the Spearman test (Figure-3). Besides that, the linear regression model showed that in each year there is a PSA increase of 0.0055 ng / mL (β = 0.0055; r^2^ = 0.0020; p < 0.001). When plotting the “Baseline Group” on graphic model, a plateau between 23 and 32 years and a progressive rise after 33 years could be noted. Thus, the values for the ages 36 – 39 remained above the group mean (0.73 ng / mL) (Figure-4). Concerning percentiles, there were only minimal variations among ages demonstrating thus, a similar kinetics (Figure-5).

**Table 2:** Pos-hoc test of PSA according to age.

Age	p	n
21 vs 22	0.331	1756
22 vs 23	0.004*	2068
23 vs 24	0.427	2377
24 vs 25	0.344	2796
25 vs 26	0.331	3360
26 vs 27	0.171	3928
27 vs 28	0.068	4692
28 vs 29	0.173	5699
29 vs 30	0.410	6212
30 vs 31	0.257	7565
31 vs 32	0.418	9752
32 vs 33	0.072	11235
33 vs 34	0.440	12738
34 vs 35	0.040*	14478
35 vs 36	0.294	16261
36 vs 37	0.090	18036
37 vs 38	0.005*	20382
38 vs 39	0.490	25450

* post-hoc test Dunn test p<0.05.

**Figure 6:**
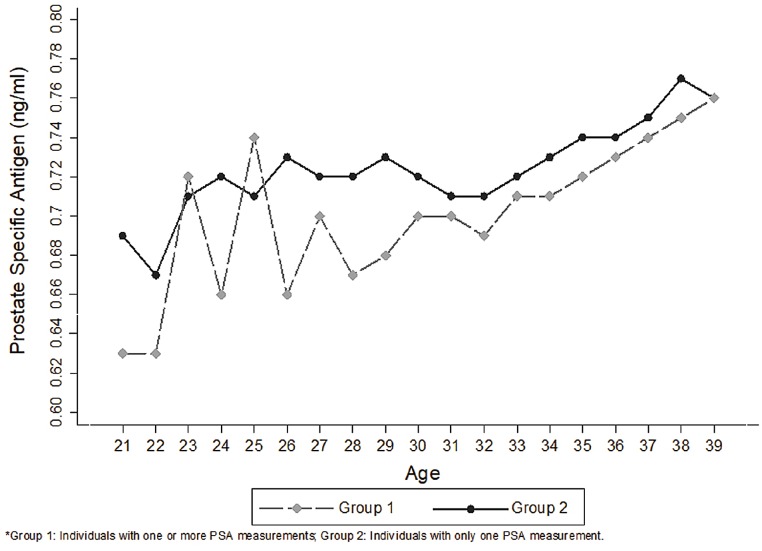
Graphic model comparing Groups 1 and 2 curves.

**Figure 7:**
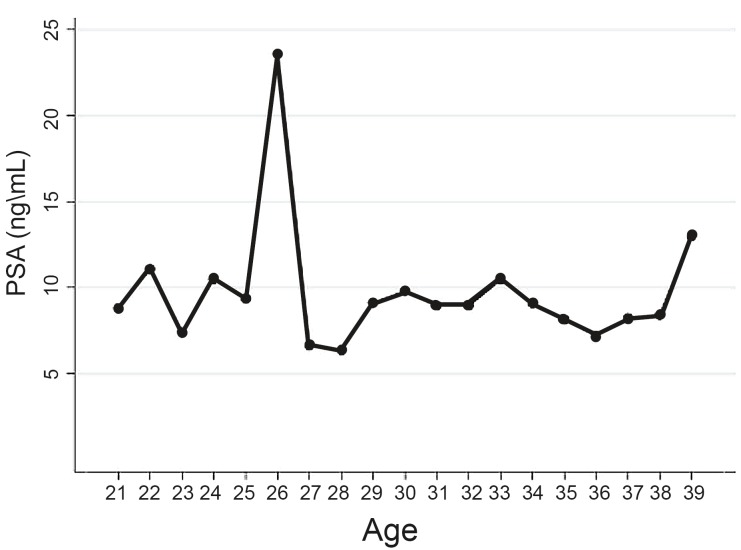
Separate analysis of those with PSA > 4.0 ng/mL.

When comparing “Group 1” and “Group 2”, the number of men with baseline PSA > 1.0 ng / mL was higher in last group (17.6% vs. 17.3%; p < 0.001) and graph curves presented a similar trend (Table-3 and Figure-6). In a separate analysis, considering the remainder with PSA > 4.0 ng / mL, we found 596 individuals with a mean age of 33.8 years and mean PSA of 9.0 ng / mL (Figure-7).

**Table 3:** Descriptive analysis of Groups 1 and 2.

Age	n (%)		Mean (SD)		Percentiles 25;75		Percentiles 90;95	
	Group 1	Group 2	Group 1	Group 2	Group 1	Group 2	Group 1	Group 2
21	143(0.44)	657(1.09)	0.63 (0.54)	0.69 (0.39)	0.32; 0.75	0.44; 0.85	0.99; 1.5	1.2; 1.4
22	200(0.61)	756(1.25)	0.63 (0.38)	0.67 (0.37)	0.39; 0.73	0.42; 0.84	1.4; 2.2	1.1; 1.3
23	220(0.67)	902(1.50)	0.72 (0.5)	0.71 (0.41)	0.39; 0.86	0.45; 0.88	1.2; 1.6	1.2; 1.5
24	277(0.85)	988(1.64)	0.66 (0.66)	0.72 (0.44)	0.41; 0.8	0.45; 0.87	1.2; 1.5	1.2; 1.5
25	307(0.94)	1224(2.03)	0.74 (0.51)	0.71 (0.45)	0.43; 0.87	0.42; 0.87	1.4; 1.5	1.2; 1.5
26	407(1.24)	1422(2.36)	0.66 (0.43)	0.73 (0.46)	0.40; 0.81	0.44; 0.90	1.1; 1.4	1.2; 1.5
27	496(1.52)	1603(2.66)	0.7 (0.44)	0.72 (0.4)	0.41; 0.86	0.45; 0.90	1.3; 1.5	1.2; 1.4
28	632(1.93)	1961(3.25)	0.67 (0.41)	0.72 (0.45)	0.41; 0.82	0.44; 0.88	1.1; 1.5	1.2; 1.6
29	832(2.54)	2274(3.77)	0.68 (0.45)	0.73 (0.43)	0.40; 0.82	0.45; 0.91	1.1; 1.5	1.2; 1.5
30	1129(3.45)	2907(4.82)	0.7 (0.45)	0.72 (0.44)	0.42; 0.85	0.44; 0.88	1.2; 1.5	1.3; 1.5
31	1308(4.00)	3151(5.23)	0.7 (0.46)	0.71 (0.43)	0.41; 0.84	0.44; 0.88	1.2; 1.4	1.2; 1.5
32	1638(5.01)	3655(6.06)	0.69 (0.43)	0.71 (0.41)	0.40; 0.86	0.44; 0.88	1.2; 1.5	1.2; 1.4
33	1959(5.99)	3983 (6.61)	0.71 (0.44)	0.72 (0.42)	0.43; 0.87	0.44; 0.89	1.2; 1.5	1.2; 1.5
34	2377(7.26)	4419(7.33)	0.71 (0.46)	0.73 (0.44)	0.41; 0.87	0.45; 0.91	1.2; 1.5	1.3; 1.5
35	2740(8.37)	4942(8.20)	0.72 (0.45)	0.74 (0.43)	0.43; 0.87.	0.45; 0.91	1.2; 1.6	1.3; 1.5
36	3276(10.01)	5303(8.80)	0.73 (0.47)	0.74 (0.45)	0.44; 0.90	0.45; 0.91	1.2; 1.5	1.3; 1.5
37	3839(11.73)	5618(9.32)	0.74 (0.48)	0.75 (0.44)	0.44; 0.90	0.46; 0.91	1.3; 1.6	1.3; 1.6
38	4577(13.99)	6348(10.53)	0.75 (0.48)	0.77 (0.47)	0.45; 0.91	0.46; 0.96	1.3; 1.6	1.3; 1.7
39	6364(19.45)	8161(13.54)	0.76 (0.48)	0.76 (0.45)	0.45; 0.92	0.46; 0.94	1.3; 1.6	1.3; 1.6
Total	32721 (100)	60274 (100)	0.73 (0.47)	0.74 (0.44)	0.43; 0.89	0.45; 0.91	1.3; 1.6	1.3; 1.5

**Group 1 =** Individuals with one or more PSA measurements; **Group 2 =** Individuals with only one PSA measurement

## DISCUSSION

PSA determination before 40 years seems interesting because it not suffers yet the drawbacks related to more advanced ages. Some other studies have examined PSA levels in young men. Preston et al. (5), for example, based on 1176 samples from a military screening program, described the median PSA between 20 – 45 years. In black men, the median PSA was 0.38, 0.45, and 0.52 ng / mL at 20 – 29, 30 – 39 and 40 – 45 years respectively. Similarly, white men presented 0.38, 0.45, and 0.40 ng / mL respectively for the same age ranges (5). In addition, Mott (2005) enrolled 845 military officer students and reported a median PSA of 0.66 ng / mL in the 40s (6). In a second study, the same author described the mean PSA as 0.9 ng / mL between 30 – 59 years (7).

These previous studies included only a small number of individuals who also had a wide age range so the results were variable, impairing conclusions. In addition, there wasn't a predetermined age to perform the baseline PSA: in most studies, this refers to the time of the first test. So, these data probably have a limited role, when considering a large population.

We need to consider that several genetic factors may be involved and determine even greater variations when comparing different countries. Some single - nucleotide polymorphisms, for example, were exclusively associated with PSA levels without affecting the risk of prostate cancer (8). Moreover, in some countries, such as Brazil, to analyze the PSA by ethnicity, we should consider the miscegenation of the population (9, 10).

Concerning studies that evaluated the link between baseline PSA and prostate cancer, Angulo et al. studied the Spanish population with 40 – 49 years. They reported that PSA > 1.0 ng / mL and ≥ 1.9 ng / mL were associated with 27.38 - fold and 161.28 - fold risk of developing cancer compared with ≤ 1.0 ng / mL (11). Similarly, based on Vickers et al. (12), the European guidelines advise that men with PSA > 1.0 ng / mL at 40 years are at elevated risk of prostate cancer several decades later (13). Indeed, across distinct populations, higher baseline PSA were associated with an increased prostate cancer risk in later years. Considering our 92.995 subjects, approximately 17% presented a baseline PSA > 1.0 ng / mL. So, it seems that this criterion probably overestimates the true risk population.

Some autopsy - based studies have detected cases of prostate cancer in younger men. In this setting, Yin et al. reported 0.5% incidence of prostate cancer among men under 49 years (14). Similarly, Soos et al. (15) reported 0%, 15% and 26.6% of prostate adenocarcinoma at 18 – 30, 31 – 40 and 41 – 50 years respectively (74% were low grade lesions). Therefore, a baseline PSA obtained before 40 years could also reduce the prostate cancer influence.

Regarding PSA in subjects younger than 40 years, we didn't find studies in the literature that included as many patients as this. Some may think that our data probably refer to patients with a familiar history of prostate cancer or another prostatic pathology to perform the PSA so early. However, most patients did not repeat the dosages (only 35.18% repeated) and among those who did, a high PSA (such as > 1.0 ng / mL) was probably not the reason. Therefore, these tests were apparently collected as health check - ups provided by some companies. Another possibility to explain these so early tests could be the widespread use of finasteride to treat alopecia. Commonly, dermatologists ask PSA before initiate the medication to have a baseline value and sometimes these tests could have been repeated during the treatment.

The novelty of this study was demonstrating the kinetics of PSA before 40 years. Considering that PSA varied only 0.1 ng / mL in 19 years (growth of 0.0055 / year), it can be affirmed that this is a very slow and progressive phenomenon. This annual growth appears to be the same regardless of the assessed percentile. In other words, it seems that even those with higher PSA tend to remain stable during this period. It could be suggested that any PSA between 21 – 39 years represent the baseline value without significant distortions.

Considering the large “n” and the inclusion of all age ranges, the data obtained reflect somewhat general population and may be useful to assess this topic and advise patients. A similar tendency of curves was observed when comparing the two groups (“Group 1” and “Group 2”). This similarity reinforces the representativeness of the data in relation to the PSA kinetics and could be interpreted as a kind of internal validation.

The fact that all dosages have been carried out at one institute with the same kit may represent an advantage considering the marked diversity of PSA assay techniques used by various laboratories (16). For example, Loeb et al. tested two different PSA tests in the same serum sample. The median and mean presented a difference of 17% and 38% respectively. Furthermore, PSA differed by greater than 0.4 ng / mL in 26%, greater than 0.75 ng / mL in 14.5% and greater than 2.0 ng / mL in 4.5% of the studied population (17).

The greatest limitation of this study was the lack of clinical data which could have influence in PSA (like history of prostatitis or recent urologic procedures). Thus, the reasons for these assays in young patients were not clear, affecting the representativeness of data. Other limitations are the retrospective nature of this study and the different numbers of individuals included per age. However, considering our outcomes, we might consider evaluating all these subjects as a single group. Finally, even after analyzing the values > 4.0 ng / mL separately of our main analysis, we still have several cases of outliners at all ages. Nevertheless, they represented few patients when considered the universe of the study.

## CONCLUSIONS

Regarding baseline PSA, this study enrolled the largest number of individuals. It was demonstrated that the kinetics of PSA before 40 years is a very slow and progressive phenomenon, regardless of the assessed percentile. It could be suggested that any PSA performed in this age range could represent the baseline value without significant distortions. Finally, we found about 17% of baseline PSA > 1.0 ng / mL. Thus, although this cutoff correlates with a higher risk of prostate cancer in previous studies, it could overstate the true population at risk.
